# Smart Electrospun Hybrid Nanofibers Functionalized with Ligand-Free Titanium Nitride (TiN) Nanoparticles for Tissue Engineering

**DOI:** 10.3390/nano11020519

**Published:** 2021-02-18

**Authors:** Viraj P. Nirwan, Eva Filova, Ahmed Al-Kattan, Andrei V. Kabashin, Amir Fahmi

**Affiliations:** 1 Faculty of Technology and Bionics, Rhine-Waal University of Applied Science, Marie-Curie-Straße 1, 47533 Kleve, Germany; viraj-pratap.nirwan@hsrw.org; 2 Aix Marseille University, CNRS, LP3, 163 Ave. De Luminy, Case 917, 13288 Marseille, France; ahmed.al-kattan@univ-amu.fr (A.A.-K.); kabashin@lp3.univ-mrs.fr (A.V.K.); 3 Institute of Experimental Medicine of the Czech Academy of Sciences, Vídeňská 1083, 14220 Prague 4, Czech Republic; eva.filova@iem.cas.cz; 4 Bio-Nanophotonics Laboratory, MEPhI, Institute of Engineering Physics for Biomedicine (PhysBio), 31 Kashirskoe sh., 115409 Moscow, Russia

**Keywords:** electrospinning, pulsed laser ablation in liquids, nanofibers, polycaprolactone (PCL), TiN nanoparticles, theranostics, biocompatibility, scaffold for tissue engineering

## Abstract

Herein, we report the fabrication and characterization of novel polycaprolactone (PCL)-based nanofibers functionalized with bare (ligand-free) titanium nitride (TiN) nanoparticles (NPs) for tissue engineering applications. Nanofibers were prepared by a newly developed protocol based on the electrospinning of PCL solutions together with TiN NPs synthesized by femtosecond laser ablation in acetone. The generated hybrid nanofibers were characterised using spectroscopy, microscopy, and thermal analysis techniques. As shown by scanning electron microscopy measurements, the fabricated electrospun nanofibers had uniform morphology, while their diameter varied between 0.403 ± 0.230 µm and 1.1 ± 0.15 µm by optimising electrospinning solutions and parameters. Thermal analysis measurements demonstrated that the inclusion of TiN NPs in nanofibers led to slight variation in mass degradation initiation and phase change behaviour (T_m_). In vitro viability tests using the incubation of 3T3 fibroblast cells in a nanofiber-based matrix did not reveal any adverse effects, confirming the biocompatibility of hybrid nanofiber structures. The generated hybrid nanofibers functionalized with plasmonic TiN NPs are promising for the development of smart scaffold for tissue engineering platforms and open up new avenues for theranostic applications.

## 1. Introduction

The development of novel platforms for regenerative medicine forms one of the attractive applications of nanotechnologies and newly synthesized inorganic nanomaterials, which can be used in combination with organic matrices for tissue engineering [1,2,3]. Here, hybrid nanofibers fabricated via electrospinning can provide numerous advantages over counterparts prepared using conventional techniques, including the possibility of using bio-degradable natural or synthetic polymers [4,5] or conductive polymers such as poly(ferrocenylphosphinoboranes) (when blended with traditional synthetic polymer such as polystyrene) [6], good control of nanofiber dimensions, and capability of incorporating multiple drugs (even if they are hydrophobic) [6,7,8]. These attractive properties stimulate the development of novel nanofiber formulations and their functionalization for a variety of applications ranging from antimicrobial action to biosensing and tissue grafting [9,10,11,12,13]. 

Various polymers with varying molecular weights, biodegradability and hydrophilicity have been successfully electrospun in the form of homogenous nanofiber structures. Such nanofibers can have unique properties such as high surface area and pore size, but their functional domain remains limited. To enable new functionalities, one can decorate the nanofibers with functional agents such as inorganic nanoparticles (NPs) or biomolecules [14,15,16]. As an example, nanofibers functionalized by silver (Ag) and copper (Cu) NPs were shown to exhibit antibacterial activity and used as extracellular matrices (ECM) to promote wound healing and tissue engineering [17], while gold (Au) NPs are often used as additives to improve the mechanical properties of nanofibers, promote protein adsorption and cell adhesion, and reduce bacterial colonization [18,19,20]. However, since most inorganic nanomaterials are fabricated by chemical methods, implying the use of various reagents and stabilizing agents, they are often contaminated by hazardous by-products, complicating their applications in biological systems [21].

Pulsed laser ablation presents a “physical” alternative to fabricated nanomaterials, which are free from stabilizing agents and impurities [22,23]. This technique is based on the natural production of nanoclusters under the action of laser radiation on a solid target [24], followed by their release into a gaseous or liquid medium to form a nanostructured film [25,26] or nanoparticle solution [22,23], respectively. In this case, solutions of NPs can be stable even in a bare (ligand-free) state and contamination-free, which opens up avenues for their successful use in biological systems in vitro and in vivo [27,28]. As an example, we recently elaborated the technique of femtosecond (fs) laser ablation in water and organic media, which makes efficient control over the size characteristics of NPs from a variety of materials possible, including Au and Si NPs [29,30,31]. We also showed that the inclusion of such ligand-free Au and Si NPs in chitosan nanofibers via electrospinning can provide a very promising platform for drug delivery and tissue engineering applications [32,33,34,35]. Here, the functionalized nanofibers were shown to be stable, with uniform thickness for given electrospinning parameters, while the presence of NPs could lead to the decrease of their mean diameter. It was also important that such NPs-blended nanofibers did not cause any negative effects on cell viability. However, the employment of chitosan for the fabrication of nanofibers imposes some limitations on the electrospinning process due to its poor solubility and swelling behaviour [36]. In addition, post processing based on the neutralization of NH_3_^+^ is typically required to stabilize the nanofibers and adapt them for utilization in biological systems. 

We believe that the properties of nanoparticle-decorated hybrid nanofiber matrices can be further improved by the choice of appropriate materials as building blocks for such structures. Here, polycaprolactone (PCL) looks to be a promising polymer for the formation of nanofiber matrices due to the combination of biocompatibility and biodegradability options [37,38,39], as well as easy solubility in acetone and dichloromethane (DCM) for making electrospinning solutions. In addition, this polymer has been approved for use in the human body for controlled drug delivery, implants, etc. On the other hand, having a strong and broad plasmonic peak around 640–700 nm with a significant tail over 800 nm, even for small NPs sizes (<7 nm) [40], bare laser-synthesized titanium nitride (TiN) nanoparticles seem to be an extremely promising functional element for such hybrid nanofiber platforms. As shown in recent studies [37,38,41], TiN NPs have very low toxicity *in vitro* and *in vivo*, as well as initiating a strong photothermal therapeutic effect under near-infrared laser irradiation in the region of relative tissue transparency. It is also important that PCL is soluble in acetone, while femtosecond laser ablation makes possible the synthesis of stable solutions of TiN NPs in acetone, which simplifies their co-electrospinning.

In this work, we report on the successful electrospinning of PCL together with laser-synthesized TiN NPs at various ratios in order to fabricate a novel hierarchical hybrid nanofiber platform for tissue engineering. The obtained nanofibers were characterized by panel materials analysis techniques such as scanning electron microscopy (SEM), Fourier Transform Infrared Spectroscopy (FTIR), and thermal analysis (TGA and DSC). Nanofibers containing various concentrations of TiN NPs were further studied to compare their influence on cell growth behaviour and the overall cytotoxicity of functionalized nanofibers.

## 2. Materials and Methods 

### 2.1. Materials 

Commercial Polycaprolactone (PCL) powder (M_w_ = 80 kD) from Purasorb^®^, Corbion, Netherlands. Dichloromethane (DCM), acetone (analytical reagent grade) and ethanol from Carl Roth, Karlsruhe, Germany were used as solvents. A TiN (99.99%) pellet from GoodFellow, Cambridge, United Kingdom was used as the target for the synthesis of TiN NPs. 

### 2.2. Methods

#### 2.2.1. Laser-Ablative Synthesis of Bare TiN NPs 

Bare TiN NPs were synthesized using methods of femtosecond (fs) laser ablation of bulk TiN target in acetone, as was earlier described in [40]. Briefly, a 2.3 mm diameter beam from a Yb:KGW laser (Amplitude Systems, Pessac, France, 1025 nm, 480 fs, 10 kHz) was focused via a 75 mm lens on the surface of the hot-pressed TiN target placed at the bottom of a glass cuvette filled with acetone. The thickness of the liquid above the target was kept constant at 1cm. The concentration of nanoparticles in the solution was calculated as 0.15 mgL^−1^, as determined from target weight loss during laser ablation. 

#### 2.2.2. Preparation of Electrospun Solutions 

Electrospinning was performed using 7 different solutions, increasing the concentration of PCL until homogenous fibers were not identified in SEM micrographs. Two solutions were prepared using PCL, starting with the concentration of 8% (*w*/*v*) in DCM (3 mL) and acetone/ethanol (2 mL). A 5 mL solution was prepared for each concentration for electrospinning, as described in Table 1. Based on micrographs from SEM, 20% (*w*/*v*) PCL was chosen as the optimised concentration for functionalization with TiN NPs. Solutions of the polymer with TiN NPs at various concentrations were solubilized in DCM and acetone in the ratio of 3:2 (*v*/*v*).

#### 2.2.3. Electrospinning of PCL and Functionalized PCL Nanofibers

An electrospinning system from IME Technologies, WG Waalre, Netherlands, which had a climate control among many other parameters, was used for the fabrication of nanofibers. The system consisted of a closed chamber, enabling controlled temperature and humidity, as well as excluding the contact of users with high voltages. The setup had a horizontal configuration and included a rotating, negatively charged cylinder as the collector. Prepared solutions were transferred to 5 mL syringes attached to a PTFE tube via a Leur-Lock with blunt ended needles (internal diameter 0.8 mm), which were inserted into a spinneret. Syringes were placed on a programmable pump to control the flow rate during electrospinning. The flow rate for all solutions was fixed at 0.2 or 0.3 mL h^−1^. The collector was covered in aluminium foil and rotated at 2500 rpm. The voltages applied to the spinneret and the collector were fixed at 10 kV and −2 kV, respectively. The electrospinning process was conducted at 18 °C, in 80% relative humidity.

### 2.3. Morphological and Physicochemical Analysis

#### 2.3.1. Electron Microscopy

To characterize the size and surface morphology of electrospun nanofibers, a scanning electron microscope (JSM-IT 100 InTouchScope^TM^, Freising, Germany) was used at the accelerating voltage of 20 kV. The observation was carried out after scratching the fibers from the aluminium foil and fixing them onto the stub using carbon tape, which was attached to the stage. A DSM 982 Gemini Zeiss system (Zeiss, Jena, Germany) at the accelerating voltage of 20 kV was used to provide higher magnification micrographs after the functionalization process. ImageJ^®^ and OriginLab software were used for analysis and graphical representation of the obtained nanofibers images.

#### 2.3.2. Thermal Analysis 

Differential scanning calorimetry (DSC) (Perkin Elmer, Waltham, MA, USA) was used to characterize the thermal properties of formed nanofibers. The samples were subjected to multiple heating cycles at a rate of 10 °C min^−1^ from −70 °C to 200 °C. Briefly, 7 mg of each fiber type was sealed in an aluminium pan, while an empty pan of similar dimensions was used as a reference. The heating cycle was carried out in a nitrogen atmosphere. The degradation profile of functional nanofibers was studied using Thermogravimetric analysis (TGA) (Perkin Elmer, Waltham, MA, USA). For each sample, 7.5 mg of scratched nanofibers from aluminium foil were placed in a ceramic cuvette at a nitrogen flow rate of 20 mL min^−1^. The samples were heated at 10 °C min^−1^ from 30 °C to 700 °C.

#### 2.3.3. Fourier Transform Infrared Spectroscopy/Attenuated Total Reflection (FTIR/ATR)

A FTIR/ATR technique was applied to nanofibers and polymers in their solid state to distinguish the specific vibrational frequency of pristine nanofibers and its changes due to functionalization. FTIR/ATR spectra were recorded using a PerkinElmer Spectrum 2000 spectrometer (Perkin Elmer, Waltham, MA, USA).

#### 2.3.4. In Vitro Testing

We performed the basic cytotoxicity tests that are commonly used in in vitro studies, including metabolic activity, cell proliferation, cell adhesion and live/dead staining assays. We used mouse 3T3 fibroblasts, clone A31, which are used as a standard cell line for cytotoxicity testing.

Nanofibers were cut to circles with a diameter of 6 mm and sterilized in 70% ethanol for 30 min, washed twice with phosphate-buffered saline (PBS) and seeded with mouse 3T3 fibroblasts at a density of 25.8 × 10^3^ cells cm^−2^ and cultured in Dulbecco´s Modified Eagle´s Medium (high glucose, Sigma Aldrich, St. Louis, MO, USA), supplemented with 10% fetal bovine serum (Cat. No. 10270, Gibco, Brazil), 100 I.U. mL^−1^ penicillin, and 100 ug mL^−1^ streptomycin (Gibco, Gaithersburg, MD, USA). Four samples were seeded for each item for MTS/DNA testing, three samples for the live/dead test and three samples for DiOC6(3)/propidium iodide staining.

Metabolic activity was evaluated using CellTiter 96^®^ AQueous One Solution Cell Proliferation Assay (MTS, Promega Corporation, Durham, NC, USA) after 1, 3, 7, 10 and 15 days. Scaffolds were put into new wells with 100 μL medium and 20 μL MTS solution and incubated in a CO_2_ incubator for 2 h. Then, absorbance of 100 μL of solution was measured using an Infinite M200 Pro spectrophotometer (Tecan, San Jose, CA, USA). Scaffolds without cells were used as negative controls.

DNA amount was measured using a Quant-iT tm dsDNA assay (High sensitivity KIT Q33120, Invitrogen, Waltham, MA, USA). Fluorescence was measured using a spectrophotometer (Infinite M200 Pro, Tecan, San Jose, CA, USA) and dsDNA amount was counted from the calibration curve of standards. 

1 μg mL^−1^ DiOC6(3) staining (Cat. No. D273, Invitrogen™, Waltham, MA, USA) was used for cell visualization on day 1. After 45 min of incubation with DiOC6(3), cells were incubated with 5 μg mL^−1^ of propidium iodide for 8 min, washed with PBS and visualized using a confocal Zeiss LSM 880 Airyscan microscope at λ_exc_ = 488 nm and λ_em_ = 505–515 nm for DiOC6(3) and λ_exc_ = 561 nm and λ_em_ > 576 nm for propidium iodide.

Live/dead staining was performed using 1 μg mL^−1^ 2′,7′-Bis(2-carboxyethyl)-5(6)-carboxyfluorescein acetoxymethyl ester (BCECF-AM Cat. No. B8806, Sigma-Aldrich), which was dissolved in serum-free medium and incubated with the scaffolds for 45 min. The scaffolds were then incubated with 5 μg mL^−1^ of propidium iodide (in PBS) for 8 min. Cells were visualized using a confocal microscope, Olympus FV10i, at λ_exc_ = 488 nm and λ_em_ = 505–515 nm for BCECF-AM (living cells) and λ_exc_ = 561 nm and λ_em_ > 576 nm for propidium iodide (dead cells).

### 2.4. Statistical Analysis

Statistical analysis was performed using SigmaStat 3.5 software (Systat Software, Inc., Richmond, CA, USA). One-way ANOVA and the Student–Newman–Keuls method were used for data analysis. The level of significance was set to 0.05.

## 3. Results

**Figure 1:**
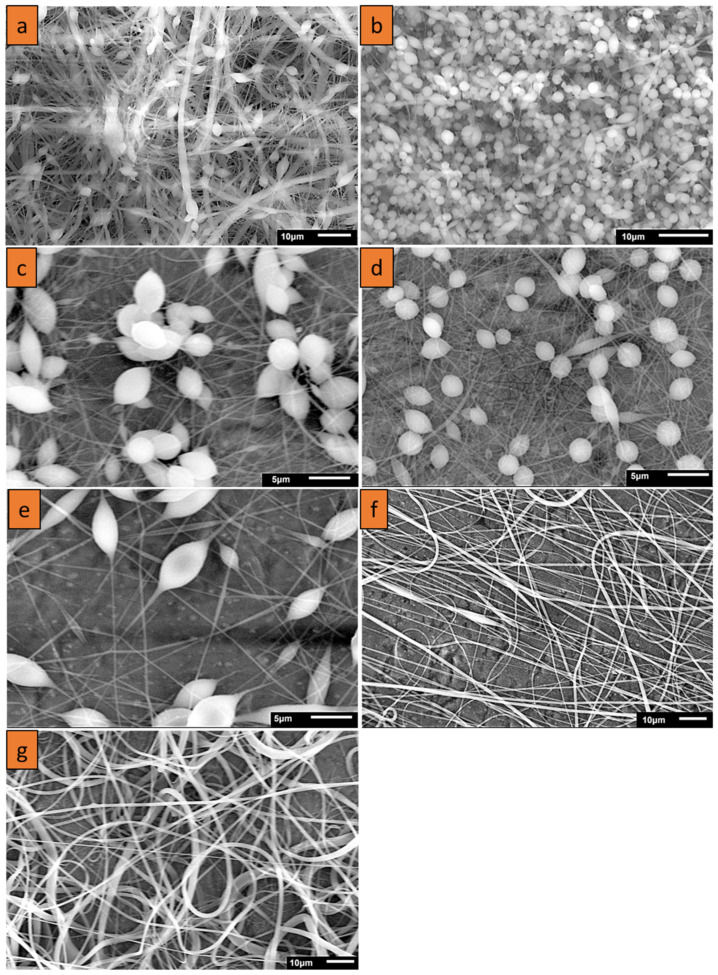
SEM micrographs of nanofibers obtained at different concentrations of PCL in nanofibers: (**a**) 8%, sample T8_0 solvent ethanol instead of acetone; (**b**) 8%, sample T8_2; (**c**) 10%, sample T10_2; (**d**) 12%, sample T12_2; (**e**) 15%, sample T15_2; (**f**) 20%, sample T20_2; (**g**) 25%, sample T25_2.

Several nanofiber formulations based on PCL solutions were prepared by electrospinning and then examined using SEM to optimize the parameters of nanofibers before their functionalization with TiN NPs. Then, the nanofibers were fabricated using various concentrations of TiN NPs. Table 1 summarizes the parameters of all the used formulations, while Figure 1 presents SEM images of the nanofibers prepared under increasing concentrations of PCL. One can see that at low concentrations of 8% (w/v), the polymer matrix presented a combination of fibers disrupted by beads, while the increase of PCL concentration led to the minimization of the number of beads. On the other hand, at the highest concentration of 25% (w/v), the fibers merged and formed structures of non-uniform thickness. In addition, the electrospinning process at high concentrations was not stable due to a high concentration of polymers blocking the flow of polymers through the spinneret. Therefore, after careful analysis of the morphologies of the obtained structures, 20% (*w*/*v*) concentration was selected as the optimal one. This concentration was later used in the co-electrospinning of PCL and TiN NPs.

**Figure 2:**
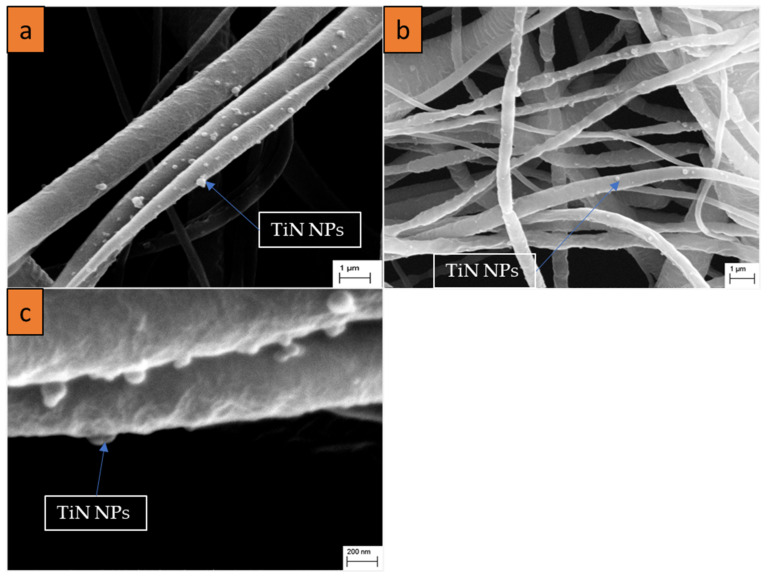
SEM micrographs of functionalized nanofibers obtained under different concentrations of TiN solubilized in solutions: (**a**) 1 mL (0.15 mg L^−1^) TiN NPs, 20% PCL (T20_1N1); (**b**) 2 mL (0.15 mg L^−1^) TiN NPs, 20% PCL (T20_0N2); (**c**) 2 mL (0.45 mg L^−1^) TiN NPs, 20% PCL (T20_0N6).

**Figure 3:**
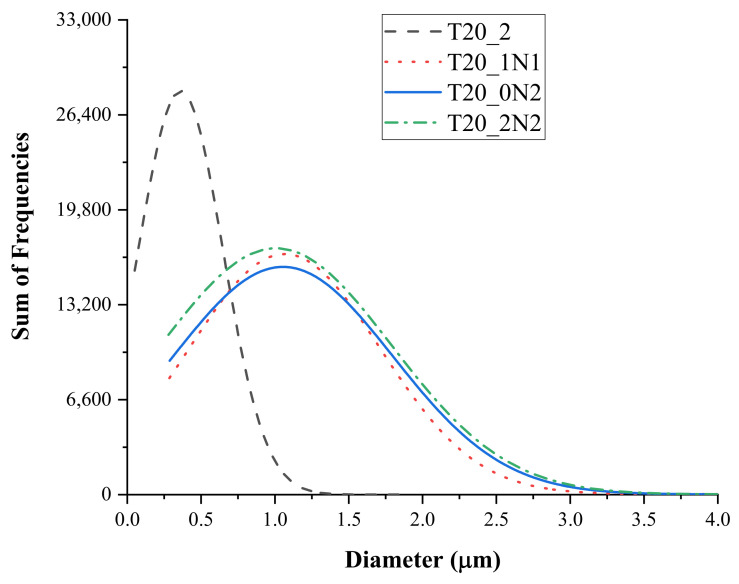
Normal diameter distribution of non-functionalized and functionalized nanofibers fabricated with different concentrations of laser-ablated TiN NPs.

Based on the optimized parameters of PCL solutions, we then performed electrospinning of PCL together with TiN NPs at different concentrations. Typical SEM images of hybrid nanofibers prepared under different nanoparticle concentrations are shown in Figure 2. One can see that the nanofibers were decorated by nanoparticles, while their mean diameter was larger compared with non-functionalized nanofibers. ImageJ^®^ software [42] was used to measure the diameter of nanofibers. These tests showed that there was an increase in mean diameter from 400 nm ± 210 nm to 1.1 µm ± 192 nm under the co-electrospinning of PCL together with NPs, while the diameters of the formed nanofibers did not depend on the concentration of TiN NPs (Figure 3 and Table 2). However, the standard deviation for the diameters of functionalized nanofibers was lower compared with the reference sample, suggesting that the nanofibers were more uniform when TiN NPs were co-electrospun with PCL, as shown in Table 2. It should be noted that the increase in nanofiber diameter with the addition of NPs could be compensated by increasing the voltage applied to the spinneret, as the increase of potential up to the threshold voltage results in a reduced diameter of the obtained nanofibers [43].

The presence of TiN NPs on the surface of PCL nanofibers can be clearly identified in SEM images, as shown in Figure 2. At high 150k resolution, single strands of nanofibers showing bunches of nanoparticles attached to the nanofiber surface could be observed (Figure 2a–c). 

### 3.1. Thermal Analysis 

**Figure 4:**
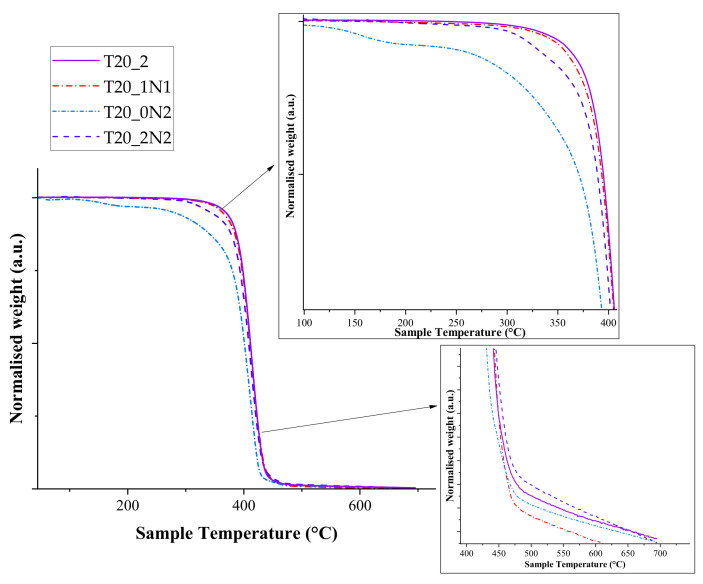
Thermogravimetric analysis (TGA) graph depicting the thermal degradation behaviour of TiN NPs-functionalized and pristine PCL nanofibers, inset zoomed view (Table 1).

Thermal analysis was carried out using TGA. Our tests did not reveal any major difference in maximal degradation temperatures and degradation initiation temperatures when pristine nanofibers and nanofibers functionalized with TiN NPs were used. All samples followed an identical trend with a single-step degradation and similar degrading temperatures. The degradation initiation temperature was calculated with a minimum of 5% mass loss for the samples (Figure 4). Pristine nanofibers displayed classical PCL degrading behaviour with mass loss initiation at 329 °C, while the functionalized nanofibers had lower initiation temperatures (328, 214 and 271 °C for T20_1N1, T20_0N2 and T20_0N6, respectively). The degradation initiation temperature of pristine nanofibers was almost identical to the one observed for functionalized nanofibers with the lowest concentration of TiN NPs (T20_1N1). However, when the temperature was further increased, both curves disassociated, while the functionalized nanofibers started to degrade faster. This trend was especially clear at the end of the temperature cycle (441 °C and 439 °C for pristine and functionalized nanofibers (T20_1N1), respectively), in which only 5% of the initial mass was present. In comparison, the end temperature for samples T20_0N2 and T20_0N6 was observed at 429 °C and 441 °C, respectively.

Sample T20_0N2, prepared under a relatively low concentration of TiN NPs (2 mL at 0.15 mg mL^−1^), started to degrade at a lower temperature of about 100 °C, which could be attributed to an insufficient number of NPs within the fibers matrix to dissipate heat. The increase in concentration of TiN NPs improved the stability of nanofibers at low temperatures due to the increase of heat capacity. Here, sample T20_0N6 with the highest concentration of TiN NPs (2 mL at 0.45 mg L^−1^) demonstrated similar degradation behaviour to pristine nanofibers. It should be also noted that most of the organic part degraded at the end of the program cycle. Indeed, about 0.1% of the initial mass was available at the cycle’s end for pristine nanofibers, while the relevant values for functionalized nanofibers were in the range of 0.2–0.35%.

**Figure 5:**
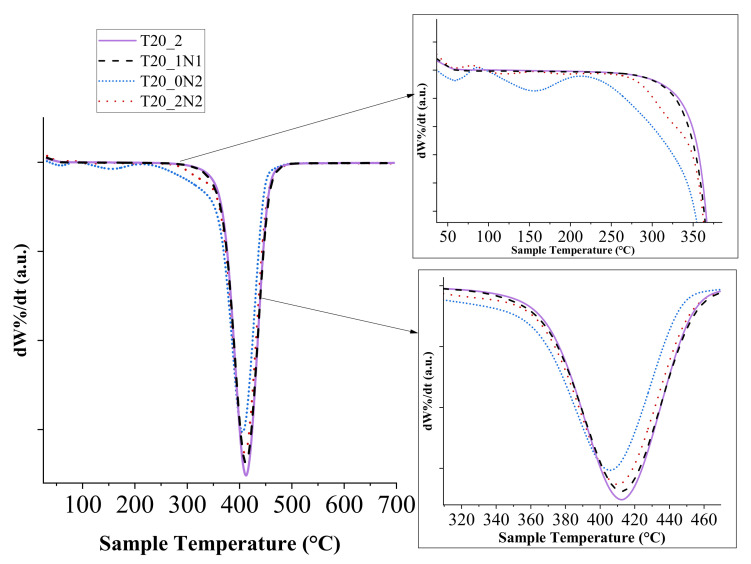
Derivative thermogram depicting the influence of TiN NPs-based functionalization on the degradation rate of PCL nanofibers, inset zoomed scale x-y. (Table 1).

As one can see from derivative thermogram (DTG) curve of the hybridized nanofibers (Figure 5), the presence of NPs at lower concentrations in the nanofibers (T20_1N1) did not affect the temperature at which the maximum mass loss event takes place, but the increase in the amount of NPs led to the decrease of this temperature by several degrees. The latter fact can be explained by the increase of heat transfer over the nanofibers due to the presence of inorganic TiN NPs in the matrix. As shown in the figure, the maximal mass loss event occurred at 412 °C for the pristine and nanofiber sample T20_1N1, while for T20_0N2 and T20_0N6 it took place at 406 °C and 409 °C, respectively.

**Figure 6:**
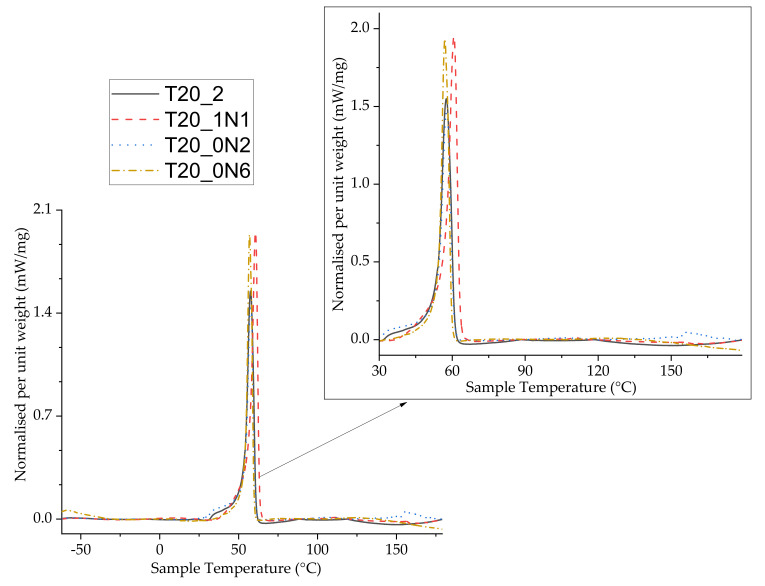
Differential scanning calorimetry graph depicting different melting temperatures within functionalized and non-functionalized PCL nanofibers, inset zoomed scale x-y. (Table 1).

Differential scanning calorimetry (DSC) tests were then undertaken to further examine the changes in thermal behaviour of functionalized nanofibers. The samples were analysed during two heating cycles, but we did not reveal differences between data from these cycles. DSC data from the first heating cycle are shown in Figure 6. Typical DSC curves for all examined samples are shown in Figure 6. Here, one can see that the peaks were endothermic without mass loss for pristine and functionalized nanofiber samples, indicating the melting phase of PCL. We observed slight differences between peaks for pristine and functionalized nanofibers. Here, phase change (melting temperature, T_m_) for pristine nanofibers and sample T20_1N1 was observed at 57.5 and 59 °C, respectively. The area under the peak showing enthalpy change (ΔH) was measured at 9.5 mWmg^−1^ for pristine nanofibers, while nanofibers functionalized at the lowest concentration of TiN NPs (T20_1N1) demonstrated a very slight increase in enthalpy up to 11.4 mWmg^−1^ (Figure 6). Nanofibers functionalized with TiN NPs at higher concentrations (T20_0N2 and T20_0N6) demonstrated similar behaviour, while the T_m_ decreased slightly from 57.8 °C to 57 °C under the highest concentration of TiN NPs. The respective change in enthalpy ΔH was 8.9 mWmg^−1^ and 8.3 mWmg^−1^ for samples T20_0N2 and T20_0N6, respectively. Such a trend was consistent with results of TGA/DTG analysis. It should be noted that there was a slight dip in curves observed for all samples when the heating program goes above 120 °C, which could be attributed a change in samples’ weight due to volatilization. No other phase change behaviour was observed until the end of the program at 200 °C. 

### 3.2. FTIR/ATR Spectroscopy 

**Figure 7:**
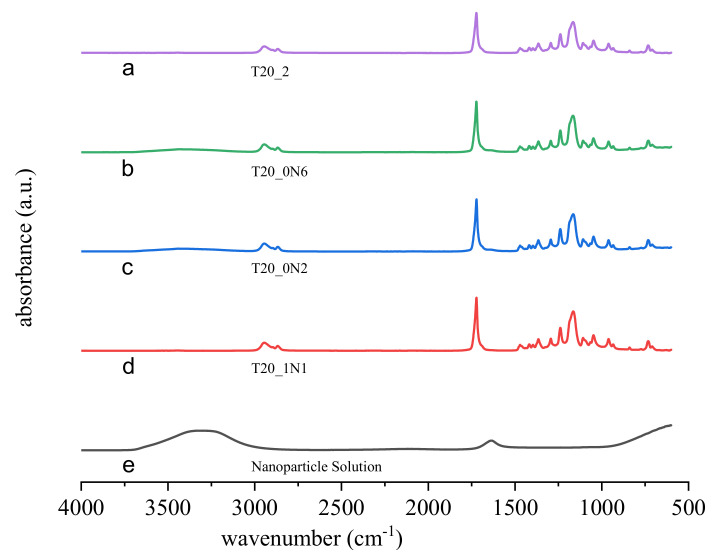
Fourier Transform Infrared Spectroscopy/Attenuated Total Reflection (FTIR/ATR) graphs of nanofiber samples prepared at different concentrations of TiN NPs. (**a**) reference fibers T20_2; (**b**) fibers prepared under 2 mL of 0.45 mg L^−1^ of TiN NPs, sample T20_0N6; (**c**) fibers prepared under 2 mL of 0.15 mg L^−1^ of TiN, sample T20_0N2; (**d**) fibers prepared under 1 mL of 0.15 mg L^−1^ of TiN NPs, T20_1N1; and (**e**) nanoparticle solution.

Functionalized and non-functionalized nanofibers were then studied using FTIR/ATR spectroscopy to analyse changes in infrared absorption peaks. Results of FTIR/ATR examination are displayed in Figure 7. As shown in the figure, the peak characterizing the presence of a nitride group could be observed at 3324 cm^−1^, which is consistent with the literature [44,45]. One can also find that the absorption spectra of the functionalized nanofibers are similar to those of the non-functionalized nanofibers. A signature peak for C=C stretching mode can be resolved at around 1650 cm^−1^, while a weak peak at 1600 cm^−1^, related to acetone, is resolvable in the case of the pure nanoparticle solution. C-O stretched bonds from 1200 to 1160 cm^−1^ are also resolvable for all samples containing PCL [46]. It is also evident that the addition of TiN NPs to the polymer matrix did not lead to interference with the absorbance peaks of PCL.

### 3.3. Biological Testing 

**Figure 8:**
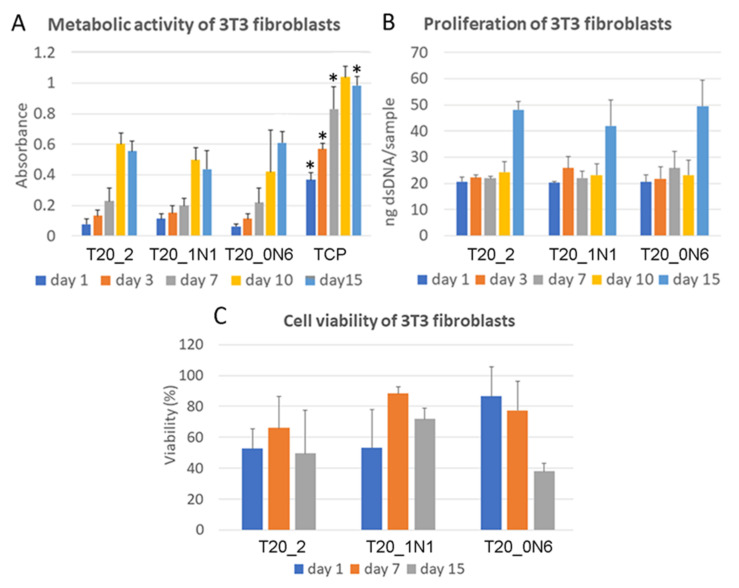
Metabolic activity measured using the MTS assay (**A**), proliferation using dsDNA assay (**B**), and viability using live/dead assay (**C**) for 3T3 fibroblasts immobilized of nanofibers with different concentrations of TiN NPs. Tissue culture plastic (TCP) was chosen as a reference to provide the highest absorbance in MTS tests. * means statistical difference related to all other samples. No significant differences were observed in either cell proliferation or cell viability tests. All assays show results as a mean and standard deviation.

In MTS assays, absorbance increased every experimental day from day 1 until day 15 for all scaffolds. As shown in Figure 8a, there were no significant changes to the metabolic activity of mouse 3T3 fibroblasts immobilized on the scaffolds from day 1 until day 10. On day 15, higher absorbance was observed for the sample prepared at a higher concentration of TiN NPs (T10). Moreover, higher absorbance compared with that of the other samples was observed for tissue culture polystyrene (TCP), which is often used to improve cell culture viability.

**Figure 9:**
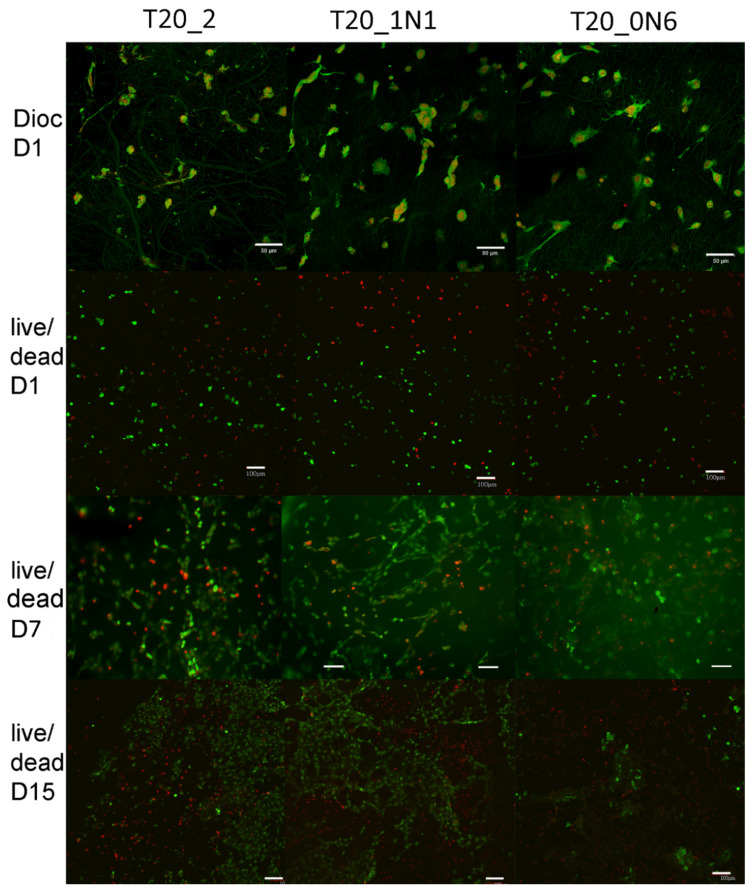
Adhesion and viability of 3T3 cells. On day 1, cell adhesion was labelled using DiOC6(3) staining (green) for plasma membranes visualization and propidium iodide staining (red) for cell nuclei (Dioc D1). As shown in the images, 3T3 cell adhered well and spread on all scaffolds. On day 1 and 15, live/dead staining was carried out using 2′,7′-Bis(2-carboxyethyl)-5(6)-carboxyfluorescein acetoxymethyl ester (BCECF-AM) staining (green viable cells) and propidium iodide (red nuclei of dead cells). Cells were visualized using the confocal microscope Zeiss LSM 880 Airyscan. Obj. 20x, bar = 50 µm for Dioc D1 staining and Obj. 10x, bar = 100 µm for live/dead staining.

As shown in Figure 8b, no significant differences were observed in dsDNA assay during the whole experiment. The amount of dsDNA increased significantly after day 10 for all samples. On day 15, the maximum amount of dsDNA was observed for both pristine and functionalized scaffolds. There is slight difference between the pristine scaffold and the scaffold functionalized with a higher concentration of TiN NPs. However, the significance is not relevant to conclude the role of TiN NPs, leading to an increase in the amount of dsDNA. On day 1, 3T3 fibroblasts were homogeneously adhered and spread over all scaffolds, while during next days, cell numbers significantly increased (Figure 9). Cell viability (live/dead assay) was evaluated from live/dead staining and microscopy analysis. Here, one could observe areas with living cells and other areas with dead cells on samples of nanofibers with TiN NPs and TCP. The presence of dead cells could be explained by contamination of nanofibers with organic solvents or inhomogeneity of nanofiber composition, which had a negative effect on cell viability. No statistical differences were observed for the different scaffolds (Figure 8c and Figure 9).

## 4. Discussion

Inorganic nanomaterials have already been explored in various clinical applications. As an example, silver NPs were found to improve wound healing in both acute burn wounds and chronic wounds [47], while zinc peroxide NPs showed both antimicrobial and anti-inflammatory effects on burn wounds [48]. The toxicity of NPs is typically influenced by chemical composition and physical characteristics, such as their size, crystalline structure, and photo-activation. In addition, the toxicity is often enhanced by the production of the reactive oxygen species (ROS) and leads to oxidative stress, inflammation, genotoxicity or carcinogenesis [49]. 

TiO_2_ nanofibers and TiO_2_ NPs in a dose range of 2.5–80 μg cm^−2^ were tested for the viability of Raw 264.7 (macrophages) and adenocarcinoma epithelial cells A549 [50]. At the highest dose, the macrophages showed a decrease in viability of 22%, but no effect was seen using A549 cells. Interestingly, NPs did not show changes in the cell viability of both cell types. Both TiO_2_ nanofibers and NPs induced low production of ROS species. TiO_2_ nanofibers changed the normal round morphology of macrophages to spindle shaped [50]. On the other hand, the anatase TiO_2_ nanospheres of longer fibre structures over 15 μm initiated an inflammatory response via alveolar macrophages and the production of inflammatory cytokines similar to silica or asbestos [51].

Owing to high wear resistance, high hardness, high scratch resistance, a low friction coefficient and high wettability, TiN-based materials have a wide spectrum of applications as coatings for articulating surfaces of implants. This material is approved by FDA and considered as physiologically inert and non-carcinogenic. Prolonged exposure of biological objects and tissues to TiN does not cause any toxic effects [52]. Titanium oxynitride coating (TiNO_x_) in thin layers prepared by atomic layer deposition on cellulose fibers demonstrated the layer depth-depending adhesion of human adipose-derived adult stem cells, with the highest adhesion occurring for samples with 20 Å coating [53]. 

Polished discs of pure titanium ASTM F67, titanium alloy (Ti-6Al-4V) ASTM F136 (10), stainless steel ASTM F138, and cobalt alloy (Co-Cr-Mo) ASTM F75 were explored through corrosion tests in conditions of bare and TiN layer-coated surfaces. 2.2 μm- height TiN coating of stainless steel increased its corrosion resistance, but no positive effect was seen on pure Ti or Ti-6Al-4V. Interestingly, the TiN coatings of these materials exhibited no cytotoxicity, intradermal irritation, or acute systemic toxicity response (measured by intraperitoneal administration of 50 mL kg^−1^ solution) [54]. 

In our experiments, TiN NPs were incorporated in PCL, a biocompatible polymer, which excludes the direct exposition of cells to potentially toxic TiN NPs. The preliminary results of the biological tests showed good biocompatibility of all tested materials, with increased metabolic activity on day 10 and the highest increase of dsDNA content on day 15. Here, T20_0N6 samples with the highest concentration of TiN NPs (4 mL) demonstrated the best metabolic activity. However, in both TiN NPs samples and PCL, some areas with dead cells were observed on day 1 and 15. As the presented in vitro tests did not show fully biocompatible material, further long-term in vitro tests over at least 5 weeks must be performed. It is important that the fabricated hybrid organic–inorganic nanofiber structures can combine good biocompatibility of PCL and specific physical properties of inorganic TiN NPs. In particular, the plasmonic and catalytic properties of TiN NPs can be used, e.g., for the phototherapy of cancer and its combination with chemotherapy and radiotherapy, which are especially effective during the G2 phase of the cell cycle and can be enhanced by optimizations of pH, microcirculation, metabolism, and the presence of sensitizers [55].

## 5. Conclusions

We have demonstrated a simple methodology to fabricate functional hybrid nanofibers based on the co-electrospinning of polycaprolactone solutions and bare laser-synthesized TiN NPs. The parameters of electrospinning using 80 kD molecular weight PCL were optimized to obtain the desired morphology and properties of the nanofibers. It was found that with 20% (w/v) concentration of PCL dissolved in dichloromethane and acetone at a 3:2 volume ratio, the fabricated nanofibers had optimal parameters for further functionalization with TiN NPs. We then fabricated hybrid nanofibers by electrospinning PCL with TiN NPs at different concentrations. Statistical analysis showed that the diameter of nanofibers became more uniform when PCL was electrospun with TiN NPs, while the presence of TiN NPs in the system was confirmed by high-resolution scanning electron microscopy, where NPs could be observed being attached to the polymer matrix or partially embedded. The examination of fiber nanofibers by DSC and TGA analysis revealed that TiN NPs provided slightly different characteristics compared with pristine nanofibers, which could be attributed to better thermal distribution within the hybrid matrix. The potential of hybrid nanofibers in biomedical applications was further assessed by biological assessment, which showed that NPs-decorated nanofibers demonstrated good biocompatibility in vitro, identical to pristine PCL nanofiber samples. The obtained results evidence that novel hybrid matrices based on PCL nanofibers and TiN NPs can serve as excellent candidates for tissue engineering, drug delivery agents and cancer theranostics.

## Figures and Tables

**Table 1:** Summary of used solutions and electrospinning parameters for successful fabrication of functionalized polycaprolactone (PCL) titanium nitride (TiN) nanofibers.

Sample Name	PCL Concentrations (*w*/*v*)	Solvents (mL)		TiN NPs	Morphology	Voltage (kV)	Flow Rate (mL h^−1^)
		DCM	Acetone				
T8_0	8%	3	0 (2 mL ethanol)	-	Fibers/Beads	10	0.3
T8_2	8%	3	2	-	Fibers/Beads	10	0.3
T10_2	10%	3	2	-	Fibers/Beads	10	0.2
T12_2	12%	3	2	-	Fibers/Beads	10	0.2
T15_2	15%	3	2	-	Fibers/Beads	10	0.2
T20_2	20%	3	2	-	Fibers	10	0.2
T25_2	25%	3	2	-	Fibers	10	0.2
T20_1N1	20%	3	1	1 mL (0.15 mg L^−1^)	Fibers	10	0.2
T20_0N2	20%	3	0	2 mL (0.15 mg L^−1^)	Fibers	10	0.2
T20_0N6	20%	3	0	2 mL (0.45 mg L^−1^)	Fibers	10	0.2

**Table 2:** Summary of diameter statistics for pristine and TiN nanoparticles (NPs) functionalized nanofibers.

Sample Name	PCL Concentrations (*w*/*v*)	TiN NPs	Mean Diameter	Standard Deviation
T20_2	20%	0	400 nm	230 nm
T20_1N1	20%	1 mL (0.15 mg L^−1^)	1.03 µm	0.19 µm
T20_0N2	20%	2 mL (0.15 mg L^−1^)	1.05 µm	0.06 µm
T20_0N6	20%	2 mL (0.45 mg L^−1^)	1.03 µm	0.15 µm

## Data Availability

Not applicable.

## References

[1] Wu T.-J., Chiu H.-Y., Yu J., Cautela M.P., Sarmento B., das Neves J., Catala C., Pazos-Perez N., Guerrini L., Alvarez-Puebla R.A., Uskoković V., Uskoković D.P. (2018). Nanotechnologies for early diagnosis, in situ disease monitoring, and prevention. Nanotechnologies in Preventive and Regenerative Medicine.

[2] Singh S., Ashfaq M., Singh R.K., Joshi H.C., Srivastava A., Sharma A., Verma N. (2013). Preparation of surfactant-mediated silver and copper nanoparticles dispersed in hierarchical carbon micro-nanofibers for antibacterial applications. N. Biotechnol..

[3] Alismail H., Du Y., Zhou J., Ryan Tian Z. A cell-sensory bioscaffold of biocompatible titanate nanofiber. Proceedings of the TechConnect Briefs 2018—Advanced Materials.

[4] Bizarria M.T.M., D’Ávila M.A., Mei L.H.I. (2014). Non-woven nanofiber chitosan/PEO membranes obtained by electrospinning. Braz. J. Chem. Eng..

[5] Gu B.K., Park S.J., Kim M.S., Kang C.M., Kim J.I., Kim C.H. (2013). Fabrication of sonicated chitosan nanofiber mat with enlarged porosity for use as hemostatic materials. Carbohydr. Polym..

[6] Lim H.S., Baek J.H., Park K., Shin H.S., Kim J., Cho J.H. (2010). Multifunctional hybrid fabrics with thermally stable superhydrophobicity. Adv. Mater..

[7] Khayet M., García-Payo C., Matsuura T. (2019). Superhydrophobic nanofibers electrospun by surface segregating fluorinated amphiphilic additive for membrane distillation. J. Memb. Sci..

[8] Blakney A.K., Ball C., Krogstad E.A., Woodrow K.A. (2013). Electrospun fibers for vaginal anti-HIV drug delivery. Antiviral Res..

[9] Nirwan V.P., Pandey S., Hey-Hawkins E., Fahmi A. (2020). Hybrid 2D nanofibers based on poly(ethylene oxide)/polystyrene matrix and poly(ferrocenylphosphinoboranes) as functional agents. J. Appl. Polym. Sci..

[10] Chen S., Cui S., Hu J., Zhou Y., Liu Y. (2017). Pectinate nanofiber mat with high absorbency and antibacterial activity: A potential superior wound dressing to alginate and chitosan nanofiber mats. Carbohydr. Polym..

[11] Hamano F., Seki H., Ke M., Gopiraman M., Lim C.T., Kim I.S. (2016). Cellulose acetate nanofiber mat with honeycomb-like surface structure. Mater. Lett..

[12] Maftoonazad N., Ramaswamy H. (2019). Design and testing of an electrospun nanofiber mat as a pH biosensor and monitor the pH associated quality in fresh date fruit (Rutab). Polym. Test..

[13] Kim J.W., Kim M.J., Ki C.S., Kim H.J., Park Y.H. (2017). Fabrication of bi-layer scaffold of keratin nanofiber and gelatin-methacrylate hydrogel: Implications for skin graft. Int. J. Biol. Macromol..

[14] Patel H., Bonde M., Srinivasan G. (2011). Biodegradable polymer scaffold for tissue engineering. Trends Biomater. Artif. Organs.

[15] Sang Y., Gu Q., Sun T., Li F., Liang C. (2008). Filtration by a novel nanofiber membrane and alumina adsorption to remove copper(II) from groundwater. J. Hazard. Mater..

[16] Cui Y., Li B., He H., Zhou W., Chen B., Qian G. (2016). Metal–Organic Frameworks as Platforms for Functional Materials. Acc. Chem. Res..

[17] Xie Z., Paras C.B., Weng H., Punnakitikashem P., Su L.-C., Vu K., Tang L., Yang J., Nguyen K.T. (2013). Dual growth factor releasing multi-functional nanofibers for wound healing. Acta Biomater..

[18] Xiang J., Li X., Ma Y., Zhao Q., Ho C.-L., Wong W.-Y. (2018). Efficient flash memory devices based on non-conjugated ferrocene-containing copolymers. J. Mater. Chem. C.

[19] Lyu J., Wang X., Liu L., Kim Y., Tanyi E.K., Chi H., Feng W., Xu L., Li T., Noginov M.A. (2016). High Strength Conductive Composites with Plasmonic Nanoparticles Aligned on Aramid Nanofibers. Adv. Funct. Mater..

[20] Ji L., Lin Z., Medford A.J., Zhang X. (2009). Porous carbon nanofibers from electrospun polyacrylonitrile/SiO2 composites as an energy storage material. Carbon N. Y..

[21] Líbalová H., Costa P.M., Olsson M., Farcal L., Ortelli S., Blosi M., Topinka J., Costa A.L., Fadeel B. (2018). Toxicity of surface-modified copper oxide nanoparticles in a mouse macrophage cell line: Interplay of particles, surface coating and particle dissolution. Chemosphere.

[22] Kabashin A.V., Delaporte P., Grojo D., Torres R., Sarnet T., Sentis M. (2010). Nanofabrication with Pulsed Lasers. Nanoscale Res. Lett..

[23] Zhang D., Gökce B., Barcikowski S. (2017). Laser synthesis and processing of colloids: Fundamentals and applications. Chem. Rev..

[24] Geohegan D.B., Puretzky A.A., Duscher G., Pennycook S.J. (1998). Time-Resolved Imaging of Gas Phase Nanoparticle Synthesis by Laser Ablation. Appl. Phys. Lett..

[25] Kabashin A.V., Meunier M., Leonelli R. (2001). Photoluminescence Characterization of Si-Based Nanostructured Films Produced by Pulsed Laser Ablation. J. Vac. Sci. Technol. B Microelectron. Nanom. Struct. Process. Meas. Phenom..

[26] Kabashin A.V., Meunier M. (2003). Laser-Induced Treatment of Silicon in Air and Formation of Si/SiOx Photoluminescent Nanostructured Layers. Mater. Sci. Eng. B.

[27] Kabashin A.V., Timoshenko V.Y. (2016). What Theranostic applications could ultrapure laser-synthesized Si nanoparticles have in cancer?. Nanomedicine.

[28] Kabashin A.V., Singh A., Swihart M.T., Zavestovskaya I.N., Prasad P.N. (2019). Laser-processed nanosilicon: A multifunctional nanomaterial for energy and healthcare. ACS Nano.

[29] Baati T., Al-Kattan A., Esteve M.A., Njim L., Ryabchikov Y., Chaspoul F., Hammami M., Sentis M., Kabashin A.V., Braguer D. (2016). Ultrapure laser-synthesized Si-based nanomaterials for biomedical applications: In vivo assessment of safety and biodistribution. Sci. Rep..

[30] Al-Kattan A., Ryabchikov Y.V., Baati T., Chirvony V., Sánchez-Royo J.F., Sentis M., Braguer D., Timoshenko V.Y., Estève M.-A., Kabashin A.V. (2016). Ultrapure Laser-Synthesized Si Nanoparticles with Variable Oxidation States for Biomedical Applications. J. Mater. Chem. B.

[31] Bailly A.-L., Correard F., Popov A., Tselikov G., Chaspoul F., Appay R., Al-Kattan A., Kabashin A.V., Braguer D., Esteve M.-A. (2019). In vivo evaluation of safety, biodistribution and pharmacokinetics of laser-synthesized gold nanoparticles. Sci. Rep..

[32] Al-Kattan A., Nirwan V.P., Popov A., Ryabchikov Y.V., Tselikov G., Sentis M., Fahmi A., Kabashin A.V. (2018). Recent advances in laser-ablative synthesis of bare Au and Si nanoparticles and assessment of their prospects for tissue engineering applications. Int. J. Mol. Sci..

[33] Al-Kattan A., Nirwan V.P., Munnier E., Chourpa I., Fahmi A., Kabashin A.V. (2017). Toward multifunctional hybrid platforms for tissue engineering based on chitosan(PEO) nanofibers functionalized by bare laser-synthesized Au and Si nanoparticles. RSC Adv..

[34] Nirwan V.P., Al-Kattan A., Fahmi A., Kabashin A.V. (2019). Fabrication of Stable Nanofiber Matrices for Tissue Engineering via Electrospinning of Bare Laser-Synthesized Au Nanoparticles in Solutions of High Molecular Weight Chitosan. Nanomaterials.

[35] Nirwan V.P., Al-Kattan A., Kabashin A., Fahmi A. Electrospun PEO/Chitosan Nanofibers Templated with Gold Nanoparticles Prepared with Laser and Wet Synthesis. Proceedings of the 2018 IEEE 8th International Conference Nanomaterials: Application & Properties (NAP).

[36] Croisier F., Jérôme C. (2013). Chitosan-based biomaterials for tissue engineering. Eur. Polym. J..

[37] Hajiali F., Tajbakhsh S., Shojaei A. (2018). Fabrication and Properties of Polycaprolactone Composites Containing Calcium Phosphate-Based Ceramics and Bioactive Glasses in Bone Tissue Engineering: A Review. Polym. Rev..

[38] Contreras-Cáceres R., Cabeza L., Perazzoli G., Díaz A., López-Romero J.M., Melguizo C., Prados J. (2019). Electrospun nanofibers: Recent applications in drug delivery and cancer therapy. Nanomaterials.

[39] Yahia S., Khalil I.A., El-Sherbiny I.M. (2019). Sandwich-Like Nanofibrous Scaffolds for Bone Tissue Regeneration. ACS Appl. Mater. Interfaces.

[40] Popov A.A., Tselikov G., Dumas N., Berard C., Metwally K., Jones N., Al-Kattan A., Larrat B., Braguer D., Mensah S. (2019). Laser- synthesized TiN nanoparticles as promising plasmonic alternative for biomedical applications. Sci. Rep..

[41] Zelepukin I.V., Popov A.A., Shipunova V.O., Tikhonowski G.V., Mirkasymov A.B., Popova-Kuznetsova E.A., Klimentov S.M., Kabashin A.V., Deyev S.M. (2021). Laser-synthesized TiN nanoparticles for biomedical applications: Evaluation of safety, biodistribution and pharmacokinetics. Mater. Sci. Eng. C..

[42] Rueden C.T., Schindelin J., Hiner M.C., DeZonia B.E., Walter A.E., Arena E.T., Eliceiri K.W. (2017). ImageJ2: ImageJ for the next generation of scientific image data. BMC Bioinform..

[43] Agarwal S., Greiner A., Wendorff J.H. (2013). Functional materials by electrospinning of polymers. Prog. Polym. Sci..

[44] Kim M., Hwang S., Yu J.-S. (2007). Novel ordered nanoporous graphitic C 3 N 4 as a support for Pt–Ru anode catalyst in direct methanol fuel cell. J. Mater. Chem..

[45] Rounaghi S.A., Vanpoucke D.E.P., Eshghi H., Scudino S., Esmaeili E., Oswald S., Eckert J. (2017). Mechanochemical synthesis of nanostructured metal nitrides, carbonitrides and carbon nitride: A combined theoretical and experimental study. Phys. Chem. Chem. Phys..

[46] Abdelrazek E.M., Hezma A.M., El-khodary A., Elzayat A.M. (2016). Spectroscopic studies and thermal properties of PCL/PMMA biopolymer blend. Egypt. J. Basic Appl. Sci..

[47] Tian J., Wong K.K.Y., Ho C.-M., Lok C.-N., Yu W.-Y., Che C.-M., Chiu J.-F., Tam P.K.H. (2007). Topical Delivery of Silver Nanoparticles Promotes Wound Healing. ChemMedChem.

[48] Ali S., Morsy R., El-Zawawy N., Fareed M., Bedaiwy M. (2017). Synthesized zinc peroxide nanoparticles (ZnO2-NPs): A novel antimicrobial, anti-elastase, anti-keratinase, and anti-inflammatory approach toward polymicrobial burn wounds. Int. J. Nanomed..

[49] Grande F., Tucci P. (2016). Titanium Dioxide Nanoparticles: A Risk for Human Health?. Mini Rev. Med. Chem..

[50] Allegri M., Bianchi M.G., Chiu M., Varet J., Costa A.L., Ortelli S., Blosi M., Bussolati O., Poland C.A., Bergamaschi E. (2016). Shape-related toxicity of titanium dioxide nanofibres. PLoS ONE.

[51] Hamilton R.F., Buford M., Xiang C., Wu N., Holian A. (2012). NLRP3 inflammasome activation in murine alveolar macrophages and related lung pathology is associated with MWCNT nickel contamination. Inhal. Toxicol..

[52] Gotman I., Gutmanas E.Y., Hunter G. (2017). 1.8 Wear-Resistant Ceramic Films and Coatings. Comprehensive Biomaterials II.

[53] Hyde G.K., McCullen S.D., Jeon S., Stewart S.M., Jeon H., Loboa E.G., Parsons G.N. (2009). Atomic layer deposition and biocompatibility of titanium nitride nano-coatings on cellulose fiber substrates. Biomed. Mater..

[54] Paschoal A.L., Vanâncio E.C., de Canale L.C.F., da Silva O.L., Huerta-Vilca D., de Motheo A.J. (2003). Metallic Biomaterials TiN-Coated: Corrosion Analysis and Biocompatibility. Artif. Organs.

[55] Baronzio G.F., Hager E.D. (2006). Hyperthermia in Cancer Treatment: A Primer.

